# Inferring mouse gene functions from genomic-scale data using a combined functional network/classification strategy

**DOI:** 10.1186/gb-2008-9-s1-s5

**Published:** 2008-06-27

**Authors:** Wan Kyu Kim, Chase Krumpelman, Edward M Marcotte

**Affiliations:** 1 Center for Systems and Synthetic Biology, Institute for Cellular and Molecular Biology, University of Texas at Austin, Speedway, Austin, Texas 78712, USA

## Abstract

The complete set of mouse genes, as with the set of human genes, is still largely uncharacterized, with many pieces of experimental evidence accumulating regarding the activities and expression of the genes, but the majority of genes as yet still of unknown function. Within the context of the MouseFunc competition, we developed and applied two distinct large-scale data mining approaches to infer the functions (Gene Ontology annotations) of mouse genes from experimental observations from available functional genomics, proteomics, comparative genomics, and phenotypic data. The two strategies — the first using classifiers to map features to annotations, the second propagating annotations from characterized genes to uncharacterized genes along edges in a network constructed from the features — offer alternative and possibly complementary approaches to providing functional annotations. Here, we re-implement and evaluate these approaches and their combination for their ability to predict the proper functional annotations of genes in the MouseFunc data set. We show that, when controlling for the same set of input features, the network approach generally outperformed a naïve Bayesian classifier approach, while their combination offers some improvement over either independently. We make our observations of predictive performance on the MouseFunc competition hold-out set, as well as on a ten-fold cross-validation of the MouseFunc data. Across all 1,339 annotated genes in the MouseFunc test set, the median predictive power was quite strong (median area under a receiver operating characteristic plot of 0.865 and average precision of 0.195), indicating that a mining-based strategy with existing data is a promising path towards discovering mammalian gene functions. As one product of this work, a high-confidence subset of the functional mouse gene network was produced — spanning >70% of mouse genes with >1.6 million associations — that is predictive of mouse (and therefore often human) gene function and functional associations. The network should be generally useful for mammalian gene functional analyses, such as for predicting interactions, inferring functional connections between genes and pathways, and prioritizing candidate genes. The network and all predictions are available on the worldwide web.

## Background

Mouse is one of the most important animal models for human biology and disease, sharing broad molecular, cellular, tissue, and physiological similarities with humans. Most mouse systems are in close enough equivalence to human for direct transfer of gene functions, and even show a frequent equivalence of precise phenotypes, such as the observed high similarity of mouse knockout strain phenotypes and human drug phenotypes [1]. In both mouse and humans, a large fraction of genes is still largely uncharacterized; of the approximately 28,000 mouse genes in the Mouse Genome Database, approximately 11,000 have no functional annotations [2]. Thus, it is of critical importance that approaches be explored for using current data to infer functions for these genes. One approach to interpreting the mouse (and therefore human) genome is to take advantage of the vast available collections of mouse functional genomics and phenotypic data in order to help identify functions of the many uncharacterized mouse genes.

When considering how to infer gene function from functional genomics, proteomics, and phenotypic data, two distinct strategies appear most viable. The first is the construction of classifiers that discover mappings from the observed features to the functional annotation of the genes. Classifiers use previously annotated genes as training examples to learn the relationships between genes' features and their annotations (for example, from the Gene Ontology (GO) Consortium [3]). The standard functional prediction approach is to create a family of simple classifiers, each one learning to predict a single GO annotation. This general strategy exploits the tendency of functional genomics data to directly correlate with (or exclude) GO annotation; for example, the presence of a proline dehydrogenase Pfam domain [4] is a strong indicator that the gene should be annotated with the GO term 'proline dehydrogenase activity' (GO:0004657).

The second general strategy is not to directly learn mappings from features to GO annotations, but instead to learn functional associations among the genes (that is, learning the probability for each gene pair to share annotations conditioned on the data), then propagate annotations via these associations. This 'guilt-by-association' strategy [5,6] is simple and pragmatic (for example, see [7-14]), given sufficient data to reconstruct a high-confidence, high-coverage association network. This approach takes advantage of the fact that many of the available types of functional genomics data naturally describe relationships between genes, rather than directly correlate with functions. For example, patterns of mRNA expression may themselves only provide weak features for learning specific GO annotations, but the tendency to share mRNA expression patterns across many experiments can be a strong indication of genes' tendency to work together, and thus to share GO annotations. Likewise, protein interactions directly specify associations, and thus fall naturally within this scheme.

Within the framework of the MouseFunc competition [15], we explored these two parallel computational strategies for discovering the functions of mouse genes. Using a limited set of functional genomics data provided for the mouse genes within MouseFunc, with gene names disguised and no sequences provided, contestants trained algorithms to learn the GO annotations provided for 90% of the mouse genes, and then made blind predictions on the 10% of genes whose functions were withheld by the organizers. These data and the structure of this contest provide a platform for assessing the relative merits, biases, and complementarities of these two computational strategies. Our submission to the contest combined a functional association network with a classification ensemble network, both of which involved several hand-tuned parameters. The analysis presented in the paper is focused on general properties of the two approaches; thus, we have repeated the contest using a simpler version of the classifier and network approaches. Using the identical subset of GO annotations employed in the MouseFunc competition, we evaluated the relative strengths of these two different approaches, tested several simple strategies for their combination, and evaluated which biological functions were well and poorly inferred. As this work represents a draft functional network of mouse genes, likely to be useful given the absence of large-scale experimental maps of mouse protein interactions or protein complexes, we also provide the network itself and search tools on a supporting website.  

## Results

### Data and task

The data provided in the context of the MouseFunc competition (described in Materials and methods and in [15]) consisted of mRNA expression data, eukaryotic and prokaryotic phlylogenetic profiles, Pfam and Interpro protein domain annotations, and Online Mendelian Inheritance in Man (OMIM) disease annotations, for a total of approximately 18 million individual observations. These data sets were provided (with widely varying coverage) for 21,603 mouse genes. At the time of the MouseFunc competition, 19,443 of those genes were supplied with labels, and of the remaining 2,160 unlabeled genes, 1,718 were designated as targets for functional prediction.

The gene annotations provided and to be predicted were terms from the GO. Roughly 5,600 distinct GO annotations have been defined for mouse genes, falling into three broad classes: 3,185 labeling biological processes (BP; that is, pathways), 1,874 labeling molecular functions (MF; that is, biochemical activities), and 566 labeling cellular components (CC; that is, subcellular locations and complexes). For the contest, 2,816 terms (1,726 BP, 763 MF, and 326 CC) were selected for prediction evaluation. These were separated into four frequency categories (3 to 10 occurrences among the MouseFunc gene set, 11 to 30 occurrences, 31 to 100 occurrences, and 101 to 300 occurrences).

Participants' functional prediction performance was evaluated by computing the quality of the predictions of the 2,816 designated GO annotations on the target genes, with the evaluation broken down by hierarchy (BP, MF, and CC) and frequency (3 to 10, 11 to 30, 31 to 100, and 101 to 300).

### Prediction strategies

Any functional prediction technique should identify some structure between features and annotations in the labeled data that allow the prediction of annotations in the unlabeled set. We examined two approaches for discovering this structure and using it to make predictions: a feature-based approach and a network-based approach. We also examined the complementarity of these two approaches and simple approaches for combining them.

The feature-based approach to this problem is conceptually straightforward: learn a mapping from the features (that is, the datasets) to the GO annotations. Each mapping (classifier) takes the responsibility for predicting presence or absence of one of the GO terms. Once trained, the 2,816 individual classifiers map the features of the unlabeled genes to 2,816 predictions regarding the presence or absence of GO annotations. As our representative of this strategy, we used a simple naïve Bayes classifier trained on all of the binary features (that is, excluding the co-expression datasets).

As a second strategy, we employed a network-based approach that operated on the assumption that genes are associated in a functional network, and that shared GO annotation indicates a functional linkage. The network approach learns functional associations by looking at every pair-wise combination of genes in the training set. When a pair of genes shares a given GO term, the features shared by that pair are taken to be indicative of the functional connection indicated by the shared annotations. The unlabeled genes are incorporated into the network by their features, and then annotations are propagated according to the observed linkages. To test this approach, we calculated a functional gene association network using a naïve Bayes strategy spanning approximately 91% to 98% (depending on major GO class) of the mouse genes (as described in the Materials and methods) and then propagated training set annotation terms through the network to calculate GO annotations for all of the held-out test set genes. We constructed two networks: a 'full' network ('network_full_') from all of the features and a 'slim' network ('network_slim_') from the same binary features used in the classifier.

Since these methods take dramatically different approaches to the functional prediction problem, it would be reasonable to investigate whether the two approaches are complementary, with one excelling in some areas and the other in others. To explore this possibility, we tested simple combinations of the results of the two approaches (network_full _and classifier), evaluating the maximum, mean, and minimum of their scores.

### Evaluation of the overall prediction accuracy

In order to measure the prediction performance, we calculated the AUC (area under the receiver operating characteristic [ROC] curve) and APR (average precision) for each GO class (BP, MF, and CC) and frequency category. AUC is appropriate for normalized, class distribution insensitive evaluations across many GO terms. APR provides a complementary evaluation, particularly for highly skewed data sets [16].

**Figure 1:**
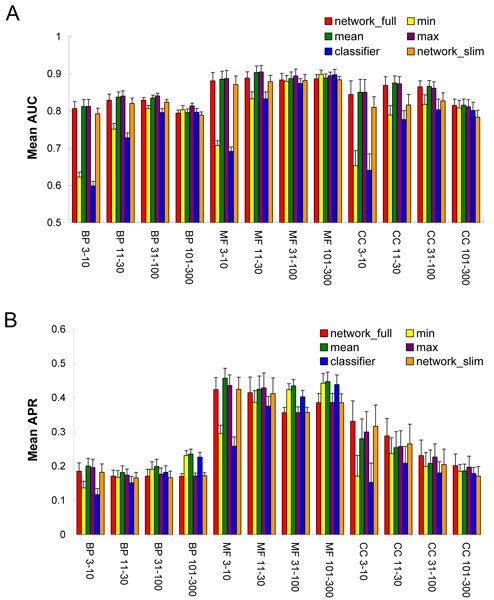
Overall performance of the various algorithms' capacity to predict mouse gene GO annotation. The performance of each general strategy ('network_full_', network-based prediction including expression data; 'network_slim_', network-based prediction excluding expression data; and 'classifier', naïve Bayes classifiers) as well as several methods of combining the network_full _and classifier scores ('mean', arithmetic mean of network and classifier scores; 'min', minimum of their scores; and 'max', maximum of their scores) is plotted as **(a) **the mean AUC and **(b) **the average APR across all Gene Ontology (GO) annotations averaged across ten-fold predictions in the indicated hierarchies (BP, biological process; CC, cellular component; MF, molecular function) and annotation specificities (terms annotating 3 to 10, 11 to 30, 31 to 100, or 101 to 300 genes). The network approach clearly outperforms the classification approach on the infrequent annotations ('3 to 10' and '11 to 30'), while the two methods perform nearly equivalently on the frequent annotations ('31 to 100' and '101 to 300'). The mean and max combinations generally perform slightly better than either of their constituents (network_full_and classifier). The full network shows a significant advantage over the slim network for CC terms and, to a lesser degree, for BP and MF terms. AUC, area under the receiver operating characteristic.

We conducted two sets of functional prediction experiments, one using ten-fold cross-validation and another repeating the MouseFunc contest. Our primary result comes from conducting the ten-fold cross-validation on all 21,603 genes of the contest. In each fold, 10% of the genes were used as a test set, and the prediction models were trained on the remaining 90%. The overall performance of each approach is plotted as average AUC and average APR across all ten folds in Figure 1. In general, we observed the network-based approach to outperform the classifier, particularly for less frequent categories (3 to 10, and 11 to 30). In more frequent categories (31 to 100, and 101 to 300), the evaluations by AUC and APR were not always consistent (Figure 1a,b). In CC, the network performed better or at least similarly in terms of both AUC and APR. For BP and MF, the classifier showed higher APR than the network, although the two approaches were similar in terms of AUC. Although the network_full _outperformed network_slim _across all GO classes and categories, the performances were not very different for both AUC and APR. Combinations of the two approaches (for example, 'mean' and 'max') generally performed the best but only slightly better than the network on its own.

The overall predictive accuracy was remarkably high, and the median AUC and APR values are summarized in Tables 1 and 2, respectively. Across the entire set of GO annotations tested, the median AUC = 0.865 and the median APR = 0.195, indicating the high power of data mining on existing datasets for the directed learning of mammalian gene functions. In addition to the ten-fold cross validation, we recreated the MouseFunc contest, using the 19,433 genes of the contest training set for training. Of the 1,718 contest target genes, we used the 1,339 annotated with at least one of the contest target GO terms. The results of this experiment are very similar to those of the cross-validation (see Additional data file 1 for the overall performance for the MouseFunc target genes).

**Table 1:** Median AUC in a ten-fold cross validation across BP, MF, CC and all the target GO annotations

	BP	MF	CC	All GO
Network_full_	0.830	0.912	0.883	0.856
Min	0.697	0.780	0.758	0.734
Mean	**0.844**	**0.926**	0.885	**0.865**
Max	0.841	0.920	**0.888**	0.863
Classifier	0.678	0.770	0.747	0.712
Network_slim_	0.819	0.905	0.834	0.842

The best prediction quality in each column is highlighted in bold. AUC, area under the receiver operating characteristic; BP, biological process; CC, cellular component; GO, Gene Ontology; MF, molecular function.

**Table 2:** Median APR in a ten-fold cross validation across BP, MF, CC and all the target GO annotations

	BP	MF	CC	All GO
Network_full_	0.114	0.388	**0.237**	0.157
Min	0.108	0.318	0.163	0.145
Mean	**0.144**	**0.431**	0.196	**0.195**
Max	0.119	0.403	0.209	0.165
Classifier	0.088	0.261	0.128	0.119
Network_slim_	0.107	0.390	0.207	0.152

The best prediction quality in each column is highlighted in bold. APR, average precision; BP, biological process; CC, cellular component; GO, Gene Ontology; MF, molecular function.

Both of the experiments exhibited consistent trends. BP was more difficult to predict than MF and CC by both approaches. The network-based approach generally outperformed the classifier based approach for infrequent annotations as measured by both AUC and APR, but the two approaches performed more similarly for frequent terms. The network approach showed more consistent performance across GO frequency categories, while the performance of the classifier approach varied more widely.

### The relative merits of the association network versus the classifier

**Figure 2:**
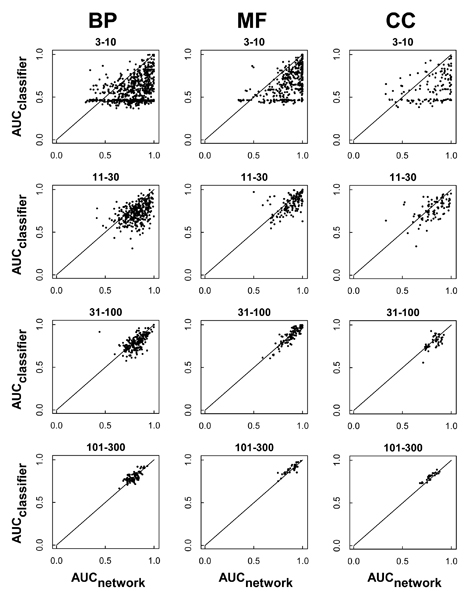
Relative performance of the network and the classifier methods by AUC. Each filled circle represents a specific Gene Ontology (GO) annotation term in the biological process (BP), cellular component (CC), or molecular function (MF) class, plotting AUC for the slim network on the x-axis and for the classifier on the y-axis. For less frequent categories ('3 to 10', '11 to 30'), AUC tends to be higher in the network_slim _than in the classifier, with most points falling under the diagonal. For more frequent terms ('31 to 100', '101 to 300'), most points are concentrated near the diagonal, suggesting that the two approaches perform similarly. This network-bias for less frequent terms was observed across all GO classes of BP, MF and CC. It is notable that for the most specific GO terms ('3 to 10'), many GO terms were predicted effectively by the network but not by the classifier with AUC_network _>> 0.5 and AUC_classifier_, although the two approaches used the same data set for training and testing. AUC, area under the receiver operating characteristic.

**Figure 3:**
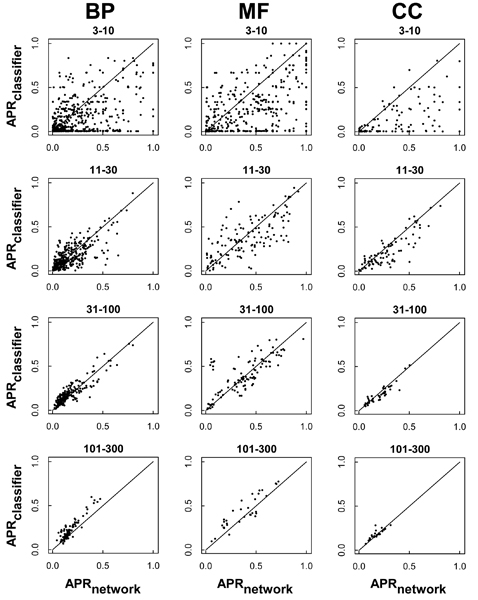
Relative performance of the network and the classifier methods by APR. Each filled circle represents a specific Gene Ontology (GO) annotation term in the biological process (BP), cellular component (CC), or molecular function (MF) class, plotting average precision (APR) for the slim network on the x-axis and for the classifier on the y-axis. The APR of the network and the classifier show a similar trend to the AUC in Figure 2. The APRs tends to be higher in the network_slim _than in the classifier for less frequent categories ('3 to 10', '11 to 30') with most points falling under the diagonal, but are similar for more frequent terms ('31 to 100', '101 to 300'), with most points near the diagonal.

Given the general dominance of the network-based approach for less frequent terms, we investigated further the relative performance of each approach. To directly compare the two approaches, we considered 'network_slim_', constructed using only the binary features in the data, which comprised exactly the same data set used to train the classifier. Figures 2 and 3 plot the relative performance of the classifier and the network_slim _for each specific GO term, separated out by GO class and frequency category. In terms of both AUC and APR, the network method performed better in the 3 to 10 and 11 to 30 frequency groups across all three hierarchies (Figures 2 and 3, respectively). In the other frequency groups (31 to 100 and 101 to 300), the points are concentrated along the diagonal, suggesting highly similar performances from both approaches. For the most specific terms (3 to 10), many GO terms show AUC_classifier _approximately 0.5 and AUC_network_slim _>> 0.5 (Figure 2). This suggests that many GO terms could be predicted effectively only by the network approach even based on the same training data, for example 'male sex differentiation' (GO:0046661), with AUC_classifier _= 0.54 and AUC_network_slim _= 0.95 (recall that AUC of approximately 0.5 indicates random prediction). In contrast, there were cases where the classifier was much better at predicting the target GO term, for example 'triplet codon-amino acid adaptor activity' (GO:0030533), with AUC_classifier _= 0.97 and AUC_network_slim _= 0.50, and 'antigen binding' (GO:0003823), with AUC_classifier _= 0.92 and AUC_network_slim _= 0.71. For MF, this classifier bias could result from a strong association with a particular domain. For example, the term 'peptide antigen binding' (GO:0042605), with AUC_classifier _= 0.87 and AUC_network_slim _= 0.64, was highly predictable by the classifier due to the annotation's strong correlation with the Pfam domain 'Perilipin family' [PF03036] involved in lipid/peptide binding. The prediction bias for network and classifier approaches is discussed in more detail in the next section.

### The network and the classifier show different, specific functional biases

**Figure 4:**
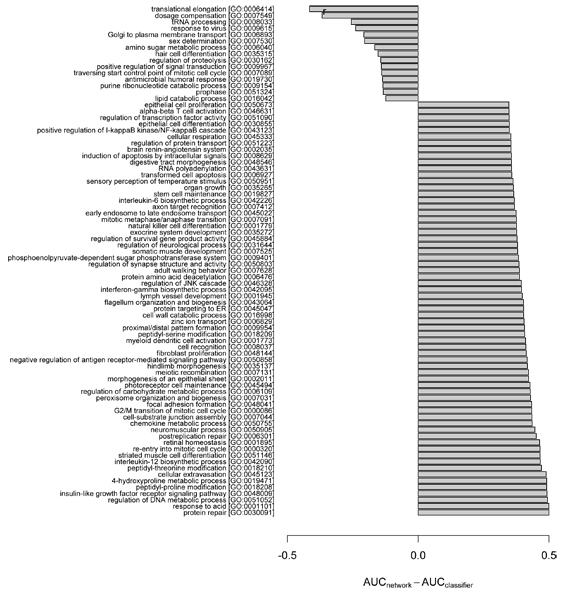
Biological process GO terms showing highly divergent predictability by the network and the classifier ensemble. Specific functional biases of biological process (BP) terms between the network and classifier ensemble are highlighted by plotting the difference between AUC_network_slim _and AUC_classifier _for GO terms showing the largest ΔAUC = max(AUC_network_, 0.5) - max(AUC_classifier_, 0.5). The GO terms with ancestor-descendant relationships were merged to the ancestor term to remove redundancy. The Gene Ontology (GO) terms with |ΔAUC| > 0.15 were plotted. As the overall performance was better in the network than in the classifier, most GO terms show positive ΔAUC. For brevity, the maximum number of terms was set to 15 and 65 for negative and positive ΔAUCs, respectively. AUC, area under the receiver operating characteristic.

**Figure 5:**
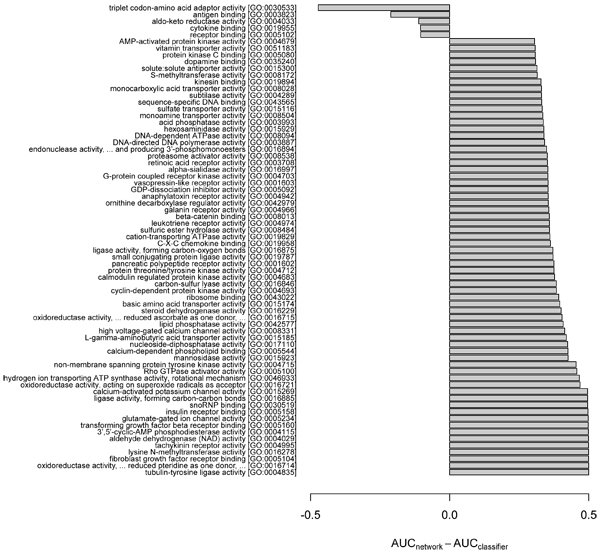
Molecular function GO terms showing highly divergent predictability by the network and the classifier ensemble. Specific functional biases of molecular function (MF) terms between the network and classifier ensemble are highlighted by plotting the difference between AUC_network_slim _and AUC_classifier _for Gene Ontology (GO) terms showing the largest ΔAUC = max(AUC_network_, 0.5) - max(AUC_classifier_, 0.5). The plot was generated using the same method as in Figure 4. AUC, area under the receiver operating characteristic.

**Figure 6:**
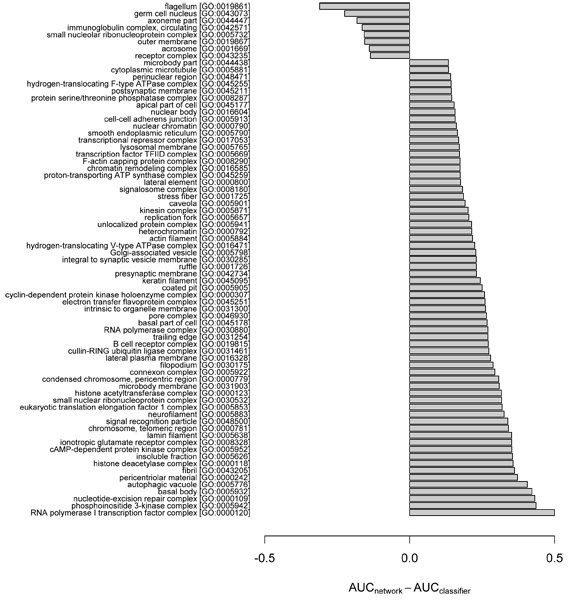
Cellular component GO terms showing highly divergent predictability by the network and the classifier ensemble. Specific functional biases of cellular component (CC) terms between the network and classifier ensemble are highlighted by plotting the difference between AUC_network_slim _and AUC_classifier _for Gene Ontology (GO) terms showing the largest ΔAUC = max(AUC_network_, 0.5) - max(AUC_classifier_, 0.5). The plot was generated using the same method as in Figure 4. GO, Gene Ontology.

Given the differences observed above between the two general strategies, we asked on which specific GO terms was there the largest difference in performance between predictions by the network and the classifier. Again, the network_slim _and the classifier were compared for consistency of training data. As AUC is insensitive to class distribution, that is, GO term frequency [17], we used AUC to measure the relative performance between the two approaches. In our investigation, we rank-ordered each GO term by the difference in AUC based on the network versus the classifier, and then examined those terms with the largest ΔAUC (Figures 4 to 6). The minimum AUC for either method was set to 0.5, thus ΔAUC values ranged from 0 to 0.5.

We observed strong differences in the predictability for some GO terms, suggesting functional biases depending on the prediction method. For example, the MF term 'triplet codon-amino acid adaptor activity' (GO:0030533) was effectively predicted by the classifier, but only poorly by the network; in contrast, the term 'fibroblast growth factor receptor binding' (GO:0005104) was predicted well by the network, but only poorly by the classifier (Figure 5). The biases do not appear confined to specific types or classes of GO terms. For example, both the network and the classifier-biased BP terms include diverse cellular functions, such as metabolism, differentiation/development and cell cycle (Figure 4). For the CC terms, the network predictions are consistent with a known ability of probabilistic gene networks to effectively capture core cellular complexes [18], such as transcription/translation apparatus, nucleotide-excision repair complex and cell signaling (Figure 6).

### Overall evaluation of well-predicted and poorly predicted mouse gene functions

We next took the best performing approach (the 'mean' combination of the network_full _and classifier) and evaluated its overall predictive performance, asking which overall biological functions were well and poorly predicted. The correlation between AUC and APR ranged from 0.55 to 0.76 for all tested GO terms, depending on the prediction algorithm. For our purpose here, AUC is chosen as the primary measure and APR as the secondary one.

**Figure 7:**
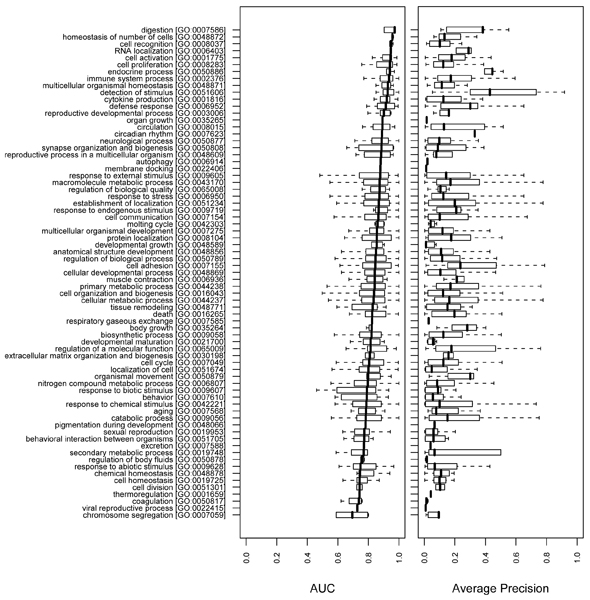
Summary of the overall performance on biological process terms. The AUC and APR distributions of high level (second level) biological process (BP) terms are plotted for the 'mean' combination of the network_full _and the classifier. The results of ten-fold cross-validation and the MouseFunc test set were merged by taking the MouseFunc test set as 11th fold. For each of the high level Gene Ontology (GO) terms, the AUC distribution (left panel) and the APR distribution (right panel) of its descendant annotations in the GO hierarchy are plotted. GO annotations are sorted from top to bottom by median AUC (bold vertical bar). Each box indicates first and third quartile; whiskers indicate minimum and maximum values. The GO terms with ancestor-descendant relationships were merged to the ancestor term to remove redundancy. APR, average precision; AUC, area under the receiver operating characteristic.

**Figure 8:**
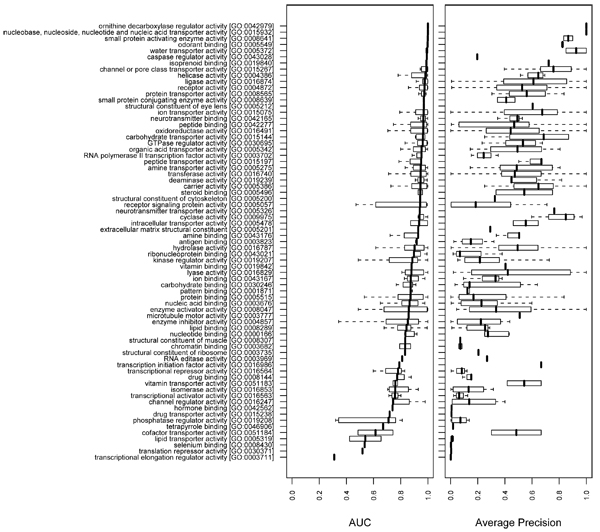
Summary of the overall performance on molecular function terms. The AUC and APR distributions of high level (second level) molecular function (MF) terms are plotted for the 'mean' combination of the network_full _and the classifier. The plot was generated in the same way as in Figure 7. APR, average precision; AUC, area under the receiver operating characteristic.

**Figure 9:**
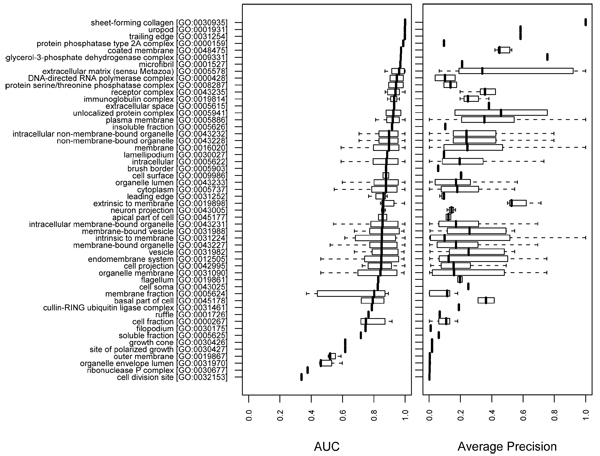
Summary of the overall performance on cellular component terms. The AUC and APR distributions of high level (second and third level) cellular component (CC) terms are plotted for the 'mean' combination of the network_full _and the classifier. The plot was generated in the same way as in Figure 7. APR, average precision; AUC, area under the receiver operating characteristic.

We employed high level GO terms, choosing terms from the second level (BP and MF) and the second and the third levels (CC) of the GO hierarchy. The GO terms in ancestor-descendant relationships were merged to the ancestor term to avoid redundancy. We examined the distribution of AUC values for all GO terms that were descendants of each high-level GO term in the hierarchy. This method allowed us to evaluate which general functions remain difficult to predict. Figures 7 to 9 plot the results, showing the distributions of AUCs and APRs as box plots rank ordered by median AUC. Both the AUCs and the APRs tend to show high variance, although the GO terms in each box plot share the same high level GO term as a more general function.

In the BP hierarchy, many of the core cellular processes are well predicted, for example 'cell proliferation' (GO:0008283), 'RNA localization' (GO:0006403), and 'cytokine production' (GO:0001816) (Figure 7). Interestingly, many GO annotations that are not single cell-based, but rather tissue specific, are also well-predicted, for example 'homeostasis of number of cells' (GO:0048872), 'immune system process' (GO:0002376), 'reproductive development process' (GO:0003006), and 'synaptic organization and biogenesis' (GO:0050808). At the lower end of performance, several other high-level GO BP terms are poorly predicted, among them 'response to abiotic stimulus' (GO:0009628), 'sexual reproduction' (GO:0019953), and 'behavioral interaction between organisms' (GO:0051705). As a wide variety of genes in many different pathways could participate in these processes and these are primarily emergent behavioral properties, it is perhaps not surprising that they are hard to learn from molecular data.

Similar trends are observed amongst MF (Figure 8) and CC categories (Figure 9), although, in general, the predictions were strongest for the MF category, with a median AUC of 0.926 across all tested MF annotations (Table 1).

### A draft association network of mouse genes

Although the primary goal of this work was to predict the functions of genes by specifically learning their GO annotation terms, the gene association network itself carries value beyond the prediction of GO terms. In particular, such networks have proven useful, especially in unicellular organisms such as yeast, for accurately predicting gene functional associations (for example [19-22]), gene essentiality [23], and the effect of gene disruption on growth rates (for example [24]). It is likely that the mouse gene network created here might have similar utility. In all, the network model is quite extensive; from the MouseFunc datasets, we estimated the probability of sharing at least one GO term for all pairs of mouse genes. The top-scoring 10% of the associations (>23 million pairs) were used to construct the association network, covering 91% to 98% of the total genes, depending on GO class. Even limiting the network to those gene pairs predicted at high confidence (*P *> 0.5) to share annotation results in a network comprising >1.6 million associations among approximately 72% to 74% of the total mouse genes. In order that this network may be used for research beyond that considered here, the complete set of links with *P *> 0.5 is made available on the supporting website [25]. Users can input a specific set of genes and retrieve additional genes most likely to operate in the same processes as the input set. This approach is thus not limited to currently annotated processes, but should be useful even for gene sets without current GO annotations for which functional relationships are captured in the network.

## Discussion

### Relative performance of the network and classifiers

We observe a significant difference in the relative performance of the two overall computational approaches. The network approach showed relatively uniform performance (AUC) across GO frequency categories, while the performance of the classifier approach showed performance positively correlated to frequency group. The network-based approach strongly outperformed the classifier approach for more specific, lower frequency GO terms. For more general (and more frequent) GO terms, the two approaches were comparable in terms of both AUC and APR. Since prediction performance is influenced by a number of factors, such the amount of data in the training genes, the data available for the test genes, the reliability of the data set, the algorithm and its parameters, and the inherent predictability of each GO term or target function, the reason for the performance differential on infrequent annotations is not obvious. We offer two plausible explanations below.

First, in the network approach, a test gene prediction is made by the transfer of GO term annotations from its network neighbors. As long as the network is more densely connected among the genes sharing a target GO term, the prediction score for the target GO term will dominate the scores for other terms. As the connectivity of a gene is independent of term frequency but is determined by the number and the types of available features, the network approach may show uniform performance across the GO frequency groups. In contrast, the classifier approach may need a certain amount of training data to effectively capture the signal for a particular function. This may explain why AUC_classifier _increased from '3 to 10' to '31 to 100' and reached a plateau between '31 to 100' and '101 to 300' for all classes of BP, MF, and CC (Figure 1a).

Second, some of the binary data represent relationships (for example, PPI, phylogenetic profiles) that are probably more useful to the network approach than to the classifier approach as the network framework is concerned with deducing relationships between genes. Thus, from these data sets, the network can infer strong associations leading to better propagation of annotation via these associations. While in principle the classification strategy should be amenable to this as well, the simple implementation practiced here may not have exploited the contribution of many weak features effectively for infrequent terms.

Given these potential explanations, it should be noted that the classifier did outperform the network on a small fraction of infrequent terms (Figures 2 and 3), and that for a subset of GO annotations the classifier complemented the network predictions (upper diagonal in Figures 2 and 3), with the result that the mean score of the two approaches generally showed a higher performance than either alone (Figure 1a,b).

### Compatibility of relational data with the network approach

Many of the functional genomic data types naturally describe relationships among genes and are thus amenable to the network-based strategy but not to the classifier approach in a straightforward way. For example, mRNA expression patterns, when gathered over many distinct experimental conditions, can strongly indicate genes belonging to the same pathways, as was noted in early analyses of DNA microarray experiments [26]; the inferred pair-wise relationships are simple to incorporate in the network framework [5]. Protein-protein interactions and phylogenetic profiles are also highly compatible with the network approach. In our experiments, network_full _(including the expression data) consistently outperformed network_slim _(excluding the expression data), supporting the inclusion of such relational data, easily accommodated in the network approach.

### Enhancement of learning rare Gene Ontology annotations

The strikingly improved performance of the network for rare (for example, level 3 to 10) GO annotations raises the question of why the network should show such an improvement for these cases. In particular, the related MouseFunc implementation of this algorithm was one of the top performers in the MouseFunc competition for the GO level 3 to 10 annotation predictions on held-out genes, and by a considerable margin in the BP and CC categories. One possibility for this enhanced performance relates to an intrinsic property of the network for allowing only functionally related genes to propagate annotation - that is, not all terms are considered as candidates for all genes, but rather only those genes directly linked to a gene carrying a term can be considered for having that term. This local connectivity produces two related effects, one on the prior odds of each annotation being awarded and one on the prior odds of each gene to receive the annotation. First, it awards terms in proportion to the prior odds of having that term - rare terms annotate only a few genes, which have limited neighbors, which in turn are the only genes considered for those terms, thereby resulting in those terms being assigned only rarely in the resulting predictions. Second, as indicated above, in addition to enforcing the low prior odds for awarding rare terms, only the linked genes can receive the rare term; not all genes have equal chances of being labeled with the term. We speculate that these dual effects may augment the network-based prediction of rare terms relative to the classification-based strategies.

### Implications for discovery of remaining gene functions

One point in particular bears noting: the data employed in the MouseFunc competition represented only a small fraction of that currently available; thus, simply by including more of the data sets already in existence, we can expect a large improvement in prediction of the remaining gene functions. In particular, there is considerable additional mRNA expression data spanning diverse mouse/human tissues, cell types, and treatments (>500 mouse and >600 human DNA microarray datasets are available in the Gene Expression Omnibus database [27], >200 mouse and >300 human SAGE libraries are available in NCBI SAGEmap [28], and approximately 600 mouse and >6,000 human DNA microarray data sets are available from the Stanford Microarray Database [29]). There are also considerable data regarding protein interactions, such as human yeast two-hybrid protein interaction data (for example [30]), omitted from MouseFunc, and considerable data from comparative genomics approaches for inferring interactions (reviewed in [31]), such as the approaches of phylogenetic profiles, gene fusions, and conserved gene neighbors.

While these latter methods do not measure protein sequence similarity, they require amino acid sequences to calculate, and therefore were excluded from the MouseFunc competition; they nonetheless add considerable value for analyzing animal gene functions. For example, knowledge of bacterial or archaeal homologs' operon organization has been shown to be a useful predictor of functional relationships among animal genes [32]; in the absence of sequence information, these data could not be included. Likewise, phylogenetic profiles based primarily upon eukaryotic genomes, as in the competition, are known to be much poorer predictors of gene functional associations than phylogenetic profiles based entirely upon bacterial genomes (for example, see [20,33]). Beyond these data, incorporation of data from other species via orthology also offers a rich source of evidence likely useful for predicting mouse gene function. We estimate that these various data (not even considering orthologous data sets) represent approximately 120 million individual experimental observations, that is, more than ten-fold more data than employed in the MouseFunc competition. Thus, there is a great opportunity for data-mining approaches of the sort explored here to directly infer mouse gene functions even if limited to current datasets.

## Conclusion

We demonstrate that mouse gene functions can be accurately learned directly from current functional genomics, proteomics, and phenotypic data sets. Using a combination of approaches, our median performance for predicting all of the held-out test set mouse gene GO annotations is reasonably high (AUC = 0.865, APR = 0.195). We explored the relative contributions to the predictions of two different computational strategies - propagation of annotation through a functional gene network and direct prediction of annotation using an ensemble of classifiers - and showed that the network-based strategy significantly outperformed the tested classifier-based strategy for relatively infrequent GO terms, while performing similarly for frequent terms. Analysis of the functions that remain poorly predicted highlights areas for improved data integration and analyses. Finally, this work also results in a single gene network that both spans the majority of mouse genes (>70% of mouse genes in the high confidence network) and is predictive of mouse (and therefore often human) gene function [25].

## Materials and methods

### Data

In the context of the MouseFunc competition, we were provided with several data sets describing features of the 21,603 genes in the mouse genome (as of February 2006). The datasets are described elsewhere [15]; we provide short descriptions here.

Three datasets, 'Zhang', 'Su', and 'SAGE', provide mRNA expression data. 'Zhang' contains 55 DNA microarray experiments covering 13,567 genes [34]. 'Su' contains 61 DNA microarray experiments covering 18,209 genes [35,36]. 'SAGE' describes SAGE tag counts at the 'quality 99' cut-off for 139 SAGE libraries in the Mouse Atlas of Gene Expression. Two datasets, 'Pfam' and 'InterPro' provide protein domain data. Pfam contains 3,133 domains covering 15,569 genes [4]. 'InterPro' contains 5,404 domains covering 16,966 genes [37]. There is some overlap between the domains represented in each dataset, and both datasets are very sparse. One dataset, 'PPI', contains protein-protein interaction data from the OPHID database [38]. 'OMIM' contains human disease gene associations for 2,488 diseases/phenotypes and covers 1,938 genes [39]; additional mouse phenotypes were available from the Mouse Genome Database [2] ('MGI') containing 33 phenotypes spanning 3,439 genes, as were very limited phylogenetic profiles calculated using BioMart [40] (18 eukaryotic species) and InParanoid [41] (21 species), covering 15,940 genes and 15,703 genes, respectively.

Along with the data sets, some GO annotations were provided for 19,443 of the 21,603 known mouse genes, although fewer than half were annotated by any one major GO branch (BP, CC, or MF). Of the remaining 2,160 genes, 1,718 were designated as the 'test set', for which functions were to be predicted. Mouse genes have been annotated with 5,624 unique GO labels; however, only 2,816 of these were used for the functional prediction problem, selected for having between 3 and 300 positive examples in the data set. Of the 2,816, 1,726 were from BP, 763 from MF, and 326 from CC. After the conclusion of the MouseFunc competition, the organizers provided the annotations of the 1,718 withheld genes. Of the 1,718, 1,339 were annotated and 379 were unannotated.

### Organization of experiments

We evaluated prediction performance in two ways: by average performance over ten-fold cross-validation, and by prediction of the annotations of the MouseFunc held-out genes. For ten-fold cross-validation, we randomly divided the 21,604 MouseFunc genes into 10 equal sized groups. Our evaluation involved ten rounds of holding out one of the groups, training the classifier and networks on the remaining groups, and predicting annotations for the held-out group. For the MouseFunc held-out genes, we trained the classifier and networks on the 19,443 annotated genes, and predicted the annotations for the 1,339 annotated MouseFunc test set genes.

### Predicting Gene Ontology annotations by a family of naïve Bayes classifiers

As a representative example of the classification approach, we trained and applied 2,815 naïve Bayes classifiers, each to predict the presence or absence of one GO annotation. Feature vectors for the classifiers were constructed from the following binary MouseFunc datasets: Pfam, InterPro, PPI, Phenotype, phylogenetic_binary, and InParanoid, totaling 11,000 binary features. Use of binary features allowed training of the naïve Bayes classifiers by simple counting. We used the log-odds form of naïve Bayes, calculated as:

$$L=\text{log}\frac{P(c=1|f)}{p(c=0|f)}=\text{log}\frac{P(c=1)}{P(c=0)}+\sum_{i=1}^{n}\text{log}P(f_{i}|c=1)-\sum_{i=1}^{n}\text{log}P(f_{i}|c=0)$$

where *c *is the class assignment (that is, 1 if the gene has the annotation, 0 if not), *f *is a given binary feature, and *i *is a counter across the features. In this form, the log-likelihood on the left hand side will be positive if the features indicate *c *= 1, and negative if the features indicate *c *= 0. Missing features were omitted from the computation. We transformed the resulting log-odds ratio into a score by projecting the log-odds ratio *L *onto a sigmoid using the following transformation:

*s *= *e*^*L*^/(1 + *e*^*L*^)

We implemented naïve Bayes and the sigmoid transformation in MATLAB (The Mathworks, Natick, MA, USA).

### Predicting Gene Ontology annotation by the association network

The network-based prediction comprised two steps: first, the probability (*P*) was predicted for each pair of genes to share a GO term using a naïve Bayes classifier based on all the available features. This produced a functional network, in which genes formed vertices, with gene pairs connected by edges of weight *P*. Second, the GO annotations of each target gene were inferred by propagating the GO annotations of the gene's network neighbors.

In calculating the network, 11 pairwise features were employed (all as provided from the MouseFunc competition): protein-protein interactions, shared interaction partners, shared phenotypes (MGI phenotypes, OMIM diseases), shared domains (Pfam, InterPro), mRNA co-expression (Zhang, Su and SAGE), and phylogenetic profiles (Ensembl, InParanoid). Each feature was scored using one of three scoring schemes: the hypergeometric probability, frequency, or Pearson correlation coefficient.

In the case of protein interactions, shared interaction partners and phylogenetic profiles, we calculated the hypergeometric probability of the partners occurring by random chance, calculated as:

$$-\text{log}P=-\text{log}\left(\sum_{i=k}^{\min(n,m)}p(i|n,m,N)\right)$$

where:

$$p(i|n,m,N)=\frac{\left(\begin{array}{l} n\\ i \end{array}\right)\left(\begin{array}{l} N-n\\ m-i \end{array}\right)}{\left(\begin{array}{l} N\\ m \end{array}\right)}$$

In this approach, *P *indicates how likely a protein pair (A, B) is to interact (or share at least *k *interaction partners, or show similar phylogenetic profiles, depending on data set) by chance. For protein-protein interactions, *n *and *m *equal the number of interactions in which each protein A and B is involved, respectively. *N *equals the total number of interactions, and *k *equals 1. In the case of shared interaction partners, *n *and *m *are as before, but *N *equals the total number of genes, and *k *equals the number of shared interaction partners between proteins A and B. For phylogenetic profiles, each profile (provided in the competition using both Ensembl and InParanoid) consisted of a binary vector of '1's and '0's, indicating the presence or absence of orthologous genes in the target organisms. In calculating the phylogenetic profile hypergeometric distribution, *n *and *m *equal the sum of each vector for the proteins A and B, respectively, *N *equals the total number of organisms used to construct the phylogenetic profiles, and *k *is the number of shared organisms in the phylogenetic profile. Finally, we calculate -*log P *as the score for each of these data sets for each pair of genes.

For the calculation of gene associations using the MGI mouse phenotype, OMIM disease, Pfam domain, and InterPro domain data sets, we employed a frequency model as follows. Each gene was considered to have a feature vector, *v*_*i*_, of length *n*, where each vector element represents the presence (1) or absence (0) of a phenotype, a disease, or a domain. We calculate *P *as the probability of sharing the observed number of vector elements by chance. -*log P *is taken as the score, calculated as:

$$-\text{log}P=\sum_{i=1}^{n}-\text{log}{\left(\frac{f_{i}}{N}\right)}^{2}$$

where *f*_*i *_is the frequency of the element *i *among the *N *total genes.

Finally, for the gene expression data sets (Zhang, Su and, SAGE), we calculated the Pearson correlation coefficient of the mRNA expression levels between each pair of genes across each of the expression data sets.

Given this set of scores associated with each pair of genes, we calculated the probability for a gene pair to share GO terms as follows. The scores for each pairwise feature were discretized into bins (9 to 28 bins, depending upon data set). Bins were chosen by ranking the gene pairs by the given score, then selecting bin locations to separate the top scoring pairs of 10,000, 20,000, 50,000, 100,000, 200,000, 500,000 ... 50,000,000 and L (the total number of pairs) gene pairs. In the case of the mRNA expression data sets, a similar process was also performed after ranking the gene pairs in reverse order (capturing anti-correlation of the expression vectors). In the event that the number of bins was less than 7 after merging the same break points, the bin size was reduced by half until the number of bins became 7 or more. For each gene pair, the posterior probability of sharing at least one GO term was then predicted using a naïve Bayesian classifier based upon the discretized bins.

Finally, a gene association network was constructed between gene pairs using the top 10% probabilities of sharing GO terms as edge weights. GO terms were predicted for each target gene by propagating the GO terms of its neighbors in the network. For a target gene with *k *neighbors (n_1_, n_2 _... n_k_), the prediction score, *S*_*i*_, for each GO term, *G*_*i*_, was calculated as:

$$S_{i}=1-\prod_{k}\left(1-P_{k}\right)$$

where *P*_*k *_is the edge weight between the target gene and its *k*-th neighbor.

### Combination of network and classifier predictions

The predicted scores from the network_full _and the classifier were combined by simply applying the given operation (minimum, maximum, mean) to each pair of predictions. If the prediction score by network is S_network _and the score by classifier is S_classifer_, then the combined score will be min(S_network_, S_classifer_), (S_network_+S_classifer_)/2 and max(S_network_, S_classifer_) for the minimum, the mean, and the maximum score, respectively.

### Evaluation of prediction performance

To evaluate the prediction performance of the various algorithms, we calculated the AUC, or area under a ROC curve, for each classification approach as applied to each GO term, and then examined the mean, median, and distribution of the AUC values. An ROC curve plots the true positive rate (sensitivity, TP/[TP+FN]) versus the false positive rate (1 - specificity, FP/(FP+TN)), where TP is true positives, FN is false negatives, FP is false positives, and TN is true negatives. A randomly guessing classifier will have an equal true positive rate and false positive rate, yielding an AUC of 0.5. A classifier making predictions better than random will produce a curve above the diagonal, and an AUC greater than 0.5, with a perfect classifier having an AUC of 1.0. A classifier making predictions worse than random will produce a curve below the diagonal, yielding an AUC between 0 and 0.5. The AUC was calculated using the ROCR library [42], which is shown to generate nearly the same AUC values as the script used for the MouseFunc competition.

We calculated the AUC for the 1,339 annotated genes (out of the 1,714 total genes) in the MouseFunc test set, using the same February 2006 GO annotations used in the competition, and the identical held-out test set of annotated genes. Summary statistics for groups of GO terms (mean, median, standard deviation) include only annotations with at least one positive example. The mean for a given group - for example, 'BP 3 to 10' - is the mean of the AUCs computed for the 1,339 annotated test set genes, for all of the GO terms belonging to the group 'BP 3 to 10', where its GO terms belong to the biological process class and the term frequency is between 3 and 10 in the MouseFunc training data set.

## Abbreviations

APR, average precision; AUC, area under a ROC curve; BP, biological process; CC, cellular component; GO, Gene Ontology; MF, molecular function; OMIM, Online Mendelian Inheritance in Man; ROC, receiver operating characteristic.

## Competing interests

The authors declare that they have no competing interests.

## Authors' contributions

WKK, CK and EMM conceived the research. WKK and CK performed the experiments. WKK, CK and EMM wrote the paper.

## Additional data file

The following additional data are available with the online version of this paper. Additional data file 1 is a figure showing overall performance for the MouseFunc target genes.

## Supplementary Material

Additional data file 1 Overall performance of the various algorithms' capacity to predict mouse gene GO annotation for the MouseFunc contest 'held out' genes. Click here for file
