# Supplementary material for: Inferring mouse gene functions from genomic-scale data using a combined functional network/classification strategy
Source: Genome Biol. 2008 Jun 27;9(Suppl 1):S5. doi: 10.1186/gb-2008-9-s1-s5 (PMC2447539; doi:10.1186/gb-2008-9-s1-s5)
Supplement: Additional data file 1 — Overall performance of the various algorithms' capacity to predict mouse gene GO annotation for the MouseFunc contest 'held out' genes. [file gb-2008-9-s1-s5-S1.pdf]

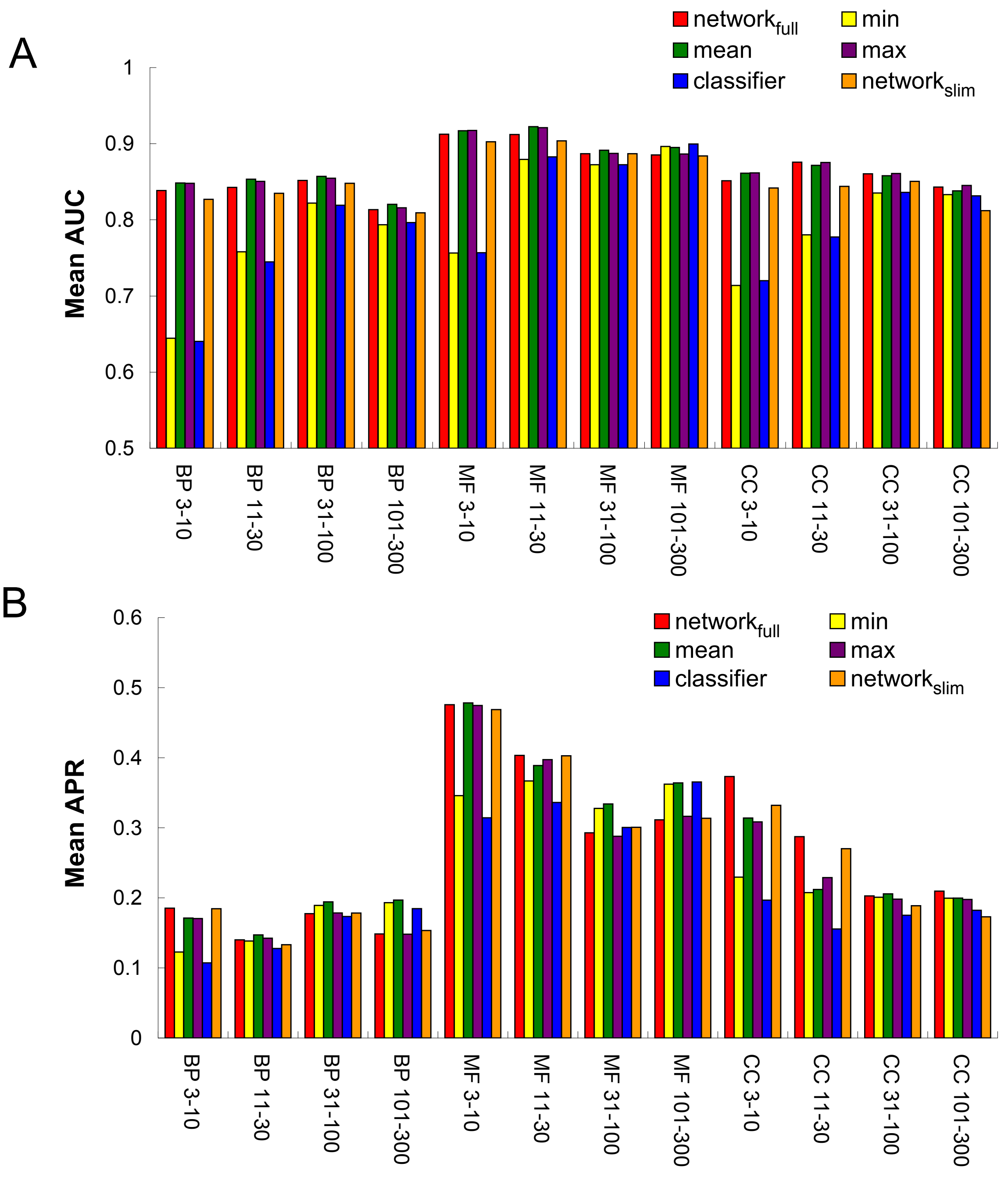

Figure S1. Overall performance of the various algorithms' capacity to predict mouse gene GO annotation for the MouseFunc contest 'held out' genes.

The performance of each general strategy ('$network_{full}$', network-based prediction including expression data; '$network_{slim}$', network-based prediction excluding expression data; 'classifier', naive Bayes classifiers; as well as several methods of combining the $network_{full}$ and classifier scores ('mean', arithmetic mean of network and classifier scores; 'min', minimum of their scores; 'max', maximum of their scores) is plotted as the mean AUC (A) and the average APR (B) across all GO annotations in the MouseFunc test set in the GO hierarchies (BP, Biological Process; CC, Cellular Component; MF, Molecular Function) and annotation specificities (terms annotating 3-10, 11-30, 31-100, or 101-300 genes). The result were consistent with the cross validation results, showing the network approach outperforming the classification approach on the infrequent annotations ('3-10' and '11-30') and similar performances on the frequent annotations ('31-100' and '101-300'). The mean and max combinations generally perform slightly better than either of their constituents ($network_{full}$ and classifier).
